# Dietary antigens drive the generation of functional cytotoxic intraepithelial lymphocytes for early defense against foodborne pathogens

**DOI:** 10.3389/fimmu.2025.1639120

**Published:** 2026-01-07

**Authors:** Jisun Jung, Jaeu Yi, Kwang Soon Kim, Charles D. Surh

**Affiliations:** 1 Department of Life Sciences, Pohang University of Science and Technology (POSTECH), Pohang, Republic of Korea; 2 Academy of Immunology and Microbiology, Institute for Basic Science (IBS), Pohang, Republic of Korea; 3 Department of Internal Medicine, Division of Rheumatology, Washington University School of Medicine, St. Louis, MO, United States; 4 Department of Biological Science, Ajou University, Suwon, Republic of Korea; 5 Division of Developmental Immunology, La Jolla Institute for Allergy and Immunology (LIAI), La Jolla, CA, United States

**Keywords:** Intraepithelial lymphocytes (IELs), dietary antigens, antigen-free mice, gut microbiota, germ-free mice, *Listeria (L.) monocytogenes*

## Abstract

**Introduction:**

Intraepithelial lymphocytes (IELs) constitute the largest lymphocyte population in the body and exhibit direct cytotoxic effector functions. Despite their abundance and importance in mucosal immunity, the mechanisms governing the generation and maintenance of functional IELs remain incompletely understood. Given the predominance of dietary components in the small intestine, dietary antigens may play a critical role in regulating the generation, maintenance, and functional maturation of IELs.

**Methods:**

To assess the relative contributions of gut microbiota and dietary antigens to IEL development and function, we analyzed IEL populations in germ-free (GF) and antigen-free (AF) mice, which are GF mice fed with an amino acid diet lacking intact dietary proteins. IEL generation, persistence, and effector function were evaluated, along with the role of IL-12 in IEL function. Resistance of foodborne pathogen was examined using Listeria monocytogenes.

**Results:**

Conventional TCRαβ+ CD4+ and CD8+ IEL populations are present in normal numbers in germ-free (GF) mice which lack microbiota. However, these IELs are severely depleted in AF mice, and the few remaining IELs in AF mice lack effector functions. Notably, while TCRαβ+ CD8αβ+ IELs in adult GF mice can persist for prolonged periods, they lose their effector function when fed with an AF diet. IL-12 presumably produced by intestinal dendritic cells plays a critical role in the maintenance of TCRαβ+ CD8αβ+ IELs and their effector functions. Importantly, mice lacking functional dietary antigen-induced TCRαβ+ CD8αβ+ IELs showed impaired early protection against oral infection with L. monocytogenes.

**Discussion:**

Collectively, these findings demonstrate that dietary antigens rather than gut microbiota, are critical for the generation of innate-like cytotoxic IELs in the small intestine. Dietary antigen-driven TCRαβ+ CD8αβ+ IELs provide rapid and local immune protection against foodborne-pathogens, highlighting a previously underappreciated role of dietary antigens in shaping intestinal immune defense.

## Introduction

The intestine harbors massive amounts of commensal microbiota and food components that are separated from the body by a single layer of epithelial cells. It also contains various innate and adaptive immune cells within the epithelium and the underlying submucosa. Despite continuous exposure to microbial and dietary antigens, the mechanisms by which the intestine maintains immune homeostasis and prevents pathogenic and chronic inflammation remain unclear. This issue is particularly critical in the small intestine, which constantly absorbs nutrients from the lumen. Unlike the colon, which is protected by a thick mucus layer, the barrier in the small intestine is relatively weaker and more susceptible to microbial and dietary antigen penetration, which could lead to infections or inflammatory conditions.

The vulnerability of the small intestine to microbial infection and dietary antigen-induced inflammation, such as allergy and celiac diseases, may explain the abundance of cytotoxic T cells in the small intestinal epithelium. These intraepithelial T lymphocytes (IELs) are dispersed evenly throughout the epithelium layer and are situated between epithelial cells (1, 2). While other immune cells also reside in the epithelium and underlying lamina propria (LP), IELs constitute the predominant lymphoid cell subset in the small intestine. Indeed, they represent the largest lymphocyte population in the body in terms of total cell numbers (3). Past works from many groups have identified five main subpopulations of IELs, namely, conventional TCRαβ^+^ (CD4^+^, CD8αβ^+^, and CD4^+^ CD8αα^+^) IELs and unconventional TCRαβ^+^ CD8αα^+^ and TCRγδ^+^ CD8αα^+^ IELs (1). Conventional IELs are derived from naïve CD4^+^ or CD8^+^ T cells following antigen encounter in mesenteric lymph nodes (mLNs) or Peyer’s patches (4, 5), whereas unconventional IELs differentiate directly from TCRαβ^+^ CD4^–^ CD8^–^ or TCRγδ^+^ CD4^–^ CD8^–^ precursors within the thymus (5).

The majority of IEL populations contain cytotoxic granules, including granzymes and perforin, in their cytoplasm (5–7). However, key questions remain regarding the precise source of antigens that drive IEL generation, the signals that induce their effector function, and the physiological function of IELs. Previous studies have investigated the influence of gut microbiota on the IEL development and effector function by comparing specific pathogen-free (SPF) and germ-free (GF) mice or through antibiotic treatment (8–10). Notably, GF mice fed with antigen-minimized diet were found to have reduced IELs with compromised cytolytic function, suggesting that dietary antigen may also play an important role in IEL development and functions (11, 12). However, direct evidence addressing the role of dietary antigens in IEL development and function remains scarce.

To directly assess the impact of dietary antigens on IEL differentiation and function, we utilized antigen-free (AF) mice, which are raised in a condition devoid of commensal microbiota and dietary antigens (13, 14). Our findings reveal that dietary antigens have a profound effect on small intestinal IELs. Specifically, the majority of conventional IELs originate from interactions with dietary antigens. In AF mice, all three populations of conventional TCRαβ^+^ (CD4^+^, CD8αβ^+^, and CD4^+^ CD8αα^+^) IELs were severely depleted while unconventional TCRαβ^+^ CD8αα^+^ IELs were partially reduced and unconventional TCRγδ^+^ IELs remain unaffected. Nonetheless, the few remaining conventional and unconventional IELs in AF mice exhibited markedly diminished effector function, consistent with previous findings (15). Our results also indicated that continuous stimulation by dietary components and interleukin-12 (IL-12) produced by dendritic cells (DCs) in the small intestinal LP are essential for the maintenance of IELs and their effector function, respectively. Importantly, dietary antigen-induced IELs, particularly conventional TCRαβ^+^ CD8αβ^+^ IELs, may function as innate-like cytotoxic IELs, providing early protection against foodborne pathogens such as *Listeria monocytogenes*.

## Materials and methods

### Mice

C57BL/6 (B6) SPF mice were maintained in our SPF animal facility. B6 mice were originally purchased from the Jackson Laboratory. GF mice and AF mice were bred in sterile isolators in our GF mouse facility at POSTECH. GF B6 breeding pairs were raised with an ultra-filtered low-molecular-weight, chemically defined elemental diet [designated as antigen-free diet (AFD)], and offspring were designated as AF mice (11, 13, 14). Hence, AF mice are GF mice fed with AFD (13). Three-week-old or 10- to 11-week-old age-matched SPF, GF, and AF mice were used for each experiment. In some cases, GF mice were fed with a commercially available amino acid diet (AAD). Detailed composition of AFD and AAD has been described previously (13, 16). Also, 6- to 10-week-old IL-15 knockout (KO) mice, IL-12Rβ2 KO mice, or CD4-dominant negative (DN) TGFβRII transgenic (Tg) mice were used with age-matched B6 mice. All animal experiments were approved and performed in accordance with ethical guidelines by the Institutional Animal Care and Use Committee of POSTECH (IACUC #POSTECH-2014-0021, #POSTECH-2016-0050, and #POSTECH-2025-0028).

### Bacteria and infections

*Listeria monocytogenes* (LM) strain 10403s carrying a recombinant internalin A with a mutation (InlA^M^) (naturally streptomycin-resistant), kindly provided by Brian S. Sheridan, was used for infection experiments. The mice were restricted from food and water for approximately 5 h prior to infection. A total of 5 × 10^8^ colony-forming units (CFU) of LM was orally infected per mouse.

### Cell preparation

The small intestine and large intestine were open longitudinally to expose the luminal side after Peyer’s patches from the small intestine were removed and then cut into 5-mm pieces. These fragments were incubated with DPBS (WelGene, Korea) containing 3% fetal calf serum (FCS) and 1 mM EDTA (WelGene) at 37°C while being stirred for 30 min. To eliminate epithelial cells, the supernatant was filtered with a 40-μm cell strainer (SPL, Korea), and suspended cells with 75% Percoll (GE Healthcare, USA) were overlaid by 40% Percoll and then centrifuged for 20 min at room temperature without brake. Afterwards, IELs were collected from the interface at a 40%–75% Percoll gradient.

### Flow cytometry

Cell suspensions were prepared and pre-blocked with anti-CD16/CD32 (93). For cell surface staining, the following fluorescent monoclonal antibodies (mAbs) were used: anti-CD45.2 (104), anti-TCRβ (H57-597), anti-TCRγδ (GL3), anti-CD4 (RM4-5), anti-CD8α (53-6.7), anti-CD8β (YTS 156.7.7), Thy1.1 (HIS51), and CD11c (N418). For intracellular staining of interferon-γ (IFN-γ) (XMG1.2), cells were stimulated by PMA and ionomycin for 4 h and then fixed and permeabilized with kits from BD Biosciences. For intracellular staining of granzyme B (GB11), T-bet (4B10), and Blimp1 (3H2-E8), cells were fixed and permeabilized with kits from BD Biosciences or eBioscience (USA). Dead cells were excluded by labeling with propidium iodide or Ghost viability dye (Tonbo, USA). Cells were stained with mAbs for 20 min at 4°C. Samples were analyzed with LSR II or FACSCanto II (BD Biosciences, USA) and analyzed by FlowJo software. To calculate the absolute number of the cell population of interest, we multiplied its frequency, determined after applying multiple gating strategies in flow cytometry, by the total leukocyte count measured using a live cell counter (Vi-Cell XR, Beckman Coulter, USA).

### Adoptively transferred OT-I cells and feeding with ovalbumin

Naïve Thy1.1^+^ OT-I CD8^+^ T cells were obtained from Thy1.1 OT-I. B6 mice or Thy1. OT-I Rag1 KO mice and adoptively transferred into non-irradiated SPF, GF, or AF B6 (Thy1.2) mice. One day after transfer, the host B6 mice were fed with 5 mg/mL ovalbumin (OVA, Sigma-Aldrich, MO, USA, grade V) in drinking water for 1 week.

### Injection of recombinant proteins

Mice were intraperitoneally (i.p.) injected with 1 μg of recombinant IL-6 (eBioscience, carrier-free), 2 μg of IL-12p70 (eBioscience, carrier-free), or 2 μg of IL-27 (eBioscience). Adult GF B6 mice were injected every 3 days for 3 weeks. Host mice adoptively transferred with OT-I cells were injected every other day for 7 days.

### Immunohistochemistry

Duodenum from SPF, GF, and AF mice was cut longitudinally and embedded in OCT compound (Sakura, USA). The tissues were cut into 5-μm sections and frozen, and the sections were fixed with methanol at −20°C. The slide sections were pre-blocked with anti-CD16/CD32 (93). For immunofluorescence staining, the slides were stained with primary anti-CD4 (GK1.5, biotin) and CD8a (53-60.7, Alexa Fluor 488) mAbs overnight at 4°C and secondary streptavidin (Alexa Fluor 594) for 2 h at room temperature. After then, the stained slides were mounted using ProLong Gold™ antifade reagent with DAPI. Confocal microscopes (Olympus and Zeiss) were used for detection of immunofluorescence.

### Quantitative RT-PCR

RNA was extracted from small intestine tissues and sorted IEL populations using TRIzol reagent (Invitrogen, USA), and cDNA was synthesized using the GoScript™ Reverse Transcriptase kit (Qiagen, USA). The *Prdm1* primers were obtained from Life Technologies. Real-time polymerase chain reaction (PCR) using TaqMan was performed on ViiA™7 (Applied Biosystems). Relative gene expression levels were normalized by the amount of the gene encoding 18s rRNA.

### RNA sequencing of small intestinal CD103^+^ CD11b^+^ dendritic cells

RNA was extracted from FACS-sorted CD103^+^ CD11b^+^ DCs from the small intestine of SPF, GF, and AF mice (*n* = 6 per group) using TRIzol reagent (Invitrogen, USA) by following the manufacturer’s procedure. CD103^+^ CD11b^+^ DCs from three mice per group were combined to obtain sufficient amount of RNA for RNA sequencing (RNA-seq). mRNA was isolated from 1 μg of RNA by using oligodT. After removal of rRNA, mRNAs were reverse-transcribed to generate single-stranded cDNA using random hexamer and reverse transcriptase, followed by double-stranded cDNA synthesis. Double-stranded cDNA was fragmented to the appropriate size and used in a standard Illumina library preparation involving end-repair, A-tailing and adapter ligation, and PCR amplification. After quantification of library using the KAPAlibrary quantification kit, RNA-seq library was sequenced on NovaSeq (Illumina, USA) followed by cluster generation. Purified total RNA and RNA-seq were performed by Theragen Etex (Korea).

### Bacterial burden counts

Two days after oral LM infection, tissues were harvested to measure infection burden. The spleen, mLNs, the entire small intestine with Peyer’s patches, and liver were ground using a 100-μm cell strainer and incubated with 0.05% Triton X-100 for 1 h at 4°C. To exclude luminal LM, entire small intestinal tissues were extensively flushed with sterile phosphate-buffered saline (PBS). The samples were plated in duplicate on brain heart infusion (BHI, MB Cell) Broth with Bacto™ agar plates added with 50 μg/mL streptomycin. Colonies were counted after 2 days at 37°C.

### Statistical analysis

Data are presented as mean ± SEM. Unpaired two-tailed Student’s *t*-test or one-way analysis of variance (ANOVA) with Tukey’s multiple comparisons test was used. *p* < 0.05 was significant. *p < 0.05, **p < 0.01, ***p < 0.001.

## Results

### Dietary antigens are crucial for the development of conventional TCRαβ^+^ IELs, less critical for unconventional TCRαβ^+^ CD8αα^+^, and not vital for TCRγδ^+^ IELs

**Figure 1:**
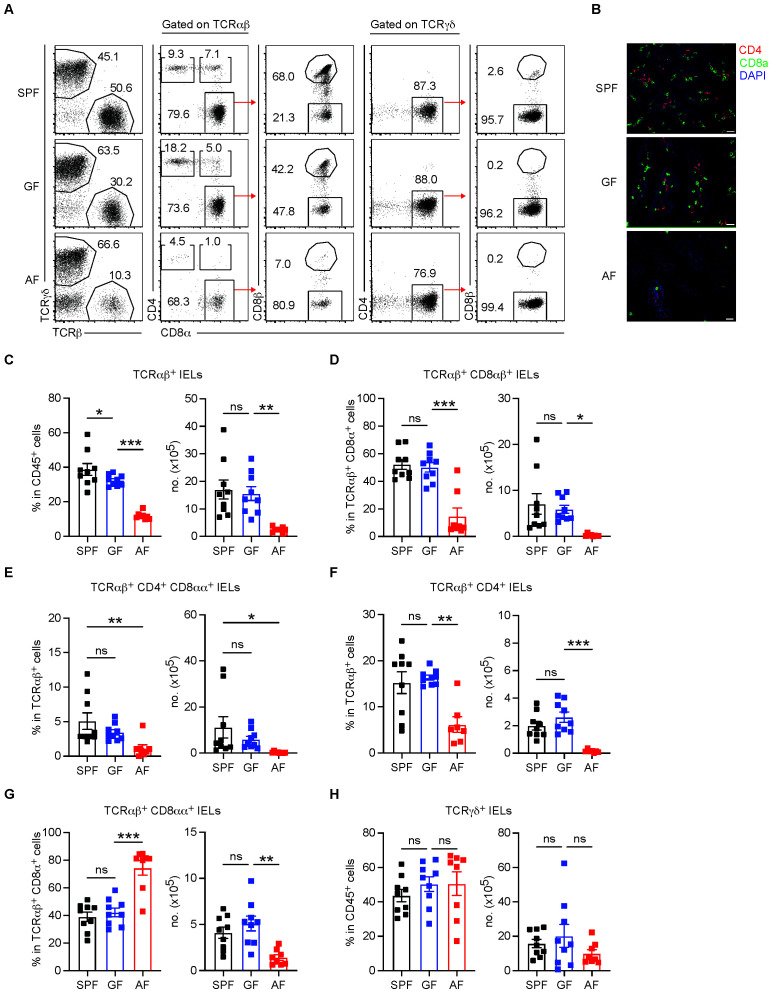
Dietary antigens are crucial for the development of conventional TCRαβ^+^ IELs, less critical for unconventional TCRαβ^+^ CD8αα^+^, and not vital for TCRγδ^+^ IELs. IELs were isolated from the small intestines of 8- to 12-week-old specific pathogen-free (SPF), germ-free (GF), and antigen-free **(AF)** mice and analyzed by flow cytometry to determine the proportions of IEL subsets. **(A)** Representative dot plots showing IEL subsets gated on lymphocytes from the indicated groups. **(B)** Immunofluorescence staining of duodenal sections showing CD4^+^ (red), CD8α^+^ (green), and nuclei (blue) in adult SPF, GF, and AF mice. Scale bars, 200 μm; magnification, 200×. **(C–H)** Percentages and total numbers of IEL subsets in SPF, GF, and AF mice. **(C)** Total TCRαβ^+^ IELs. **(D)** TCRαβ^+^ CD8αβ^+^ IELs. **(E)** TCRαβ^+^ CD4^+^ CD8αα^+^ IELs. **(F)** TCRαβ^+^ CD4^+^ IELs. **(G)** TCRαβ^+^ CD8αα^+^ IELs. **(H)** TCRγδ^+^ IELs. Data are pooled from three independent experiments (*n* = 9 per SPF and GF mice, *n* = 8 per AF mice). Statistical differences were determined by one-way ANOVA **(C–H)** with Tukey’s multiple comparisons tests. *p < 0.05, **p < 0.01, ***p < 0.001. ns, not significant. Each symbol represents an individual mouse. Error bars represent SEM.

To investigate the role of commensal microbiota and/or dietary macromolecules in IEL development, we analyzed IEL populations in the small intestinal epithelium of adult SPF, GF, and AF mice. Using an appropriate gating strategy, we distinguished all the conventional and unconventional IEL subsets (**Figure 1A**). Consistent with previous reports (17, 18), GF mice exhibited no significant reduction in the number of TCRαβ^+^ IELs compared to SPF mice (**Figures 1A–C**). The frequency and total number of conventional TCRαβ^+^ IEL subsets, including CD8αβ^+^, CD4^+^, and CD4^+^ CD8αα^+^, remained largely intact in GF mice (**Figures 1D–F**). Similarly, unconventional TCRαβ^+^ CD8αα^+^ and TCRγδ^+^ IEL subsets in GF mice were present in similar numbers to those in SPF mice, indicating a minor role of microbiota for the development of unconventional IELs in the small intestine (**Figures 1G, H**).

However, as observed in adult AF mice, deprivation of dietary macromolecules in GF mice led to a severe depletion of all conventional TCRαβ^+^ IEL subsets (**Figures 1A–F**). Immunohistological examination revealed a marked reduction of CD4^+^ and CD8^+^ IELs within the small intestinal villi of AF mice (**Figure 1B**). The total number of conventional TCRαβ^+^ IELs, including CD8αβ^+^, CD4^+^, and CD4^+^ CD8α^+^ subsets, was significantly lower in AF mice compared to SPF and GF mice (**Figures 1C–F**). In contrast, unconventional TCRαβ^+^ CD8αα^+^ IEL numbers were only partially reduced (~50%) while TCRγδ^+^ IELs remained relatively unaffected, even in AF mice (**Figures 1G, H**). These results suggest that dietary antigens are crucial for the development of conventional TCRαβ^+^ IEL subsets in the small intestine but have only a moderate effect on unconventional TCRαβ^+^ CD8αα^+^ IELs and no apparent impact on TCRγδ^+^ IELs.

### Dietary antigens are critical for the effector functions of conventional and unconventional IELs

Unlike T cells in other tissues, such as small intestinal LPs, IELs in the small intestine are poised to exert immediate effector functions upon TCR stimulation (19–21). Conventional TCRαβ^+^ IEL subsets readily produce both IFN-γ and granzyme B, whereas unconventional IELs express granzyme B and perforin following TCR stimulation (5, 22–24). Previous studies have shown that the cytolytic activity of IELs is moderately reduced in GF mice, suggesting a partial role for microbiota (8).

**Figure 2:**
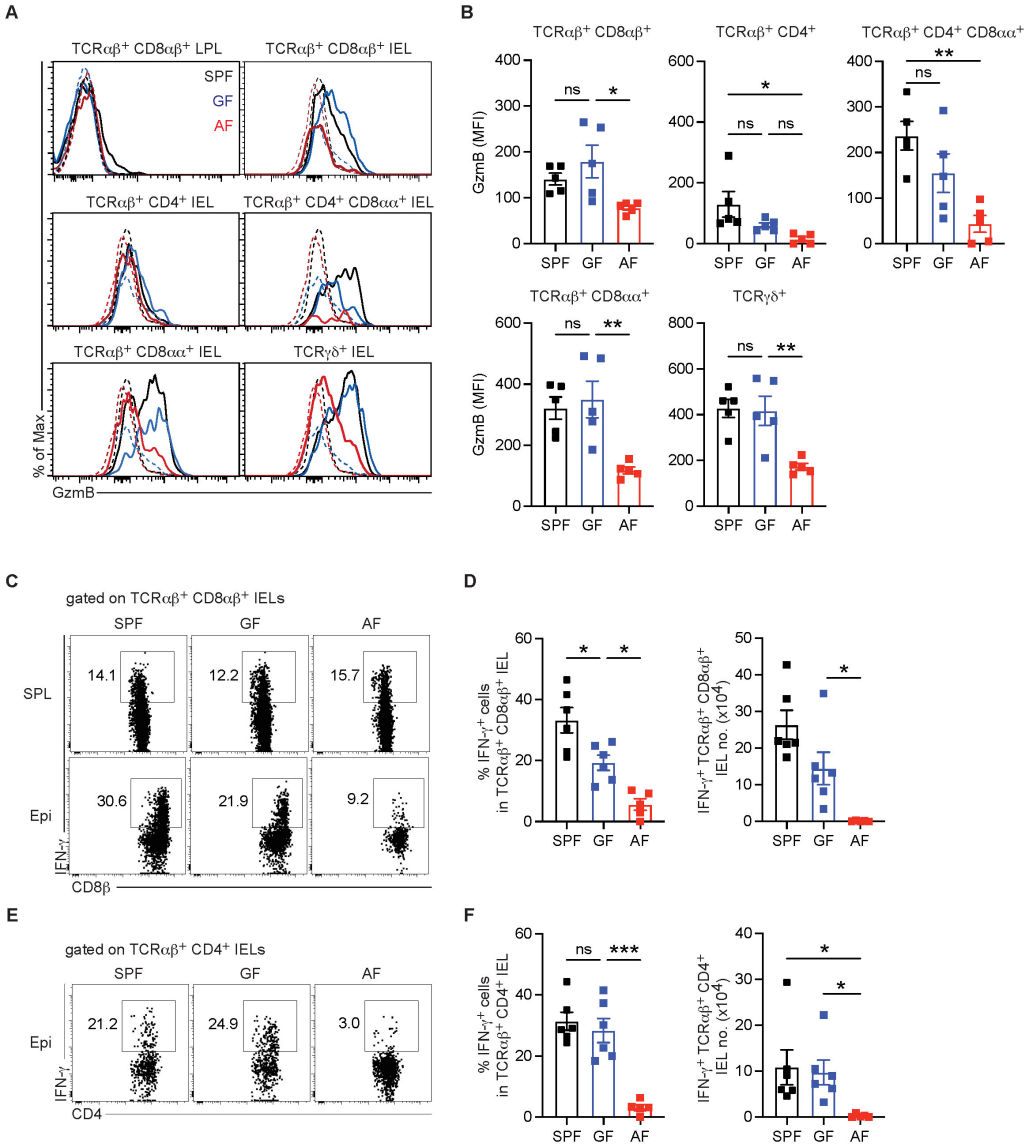
Dietary antigens are critical for the effector functions of conventional and unconventional IELs. **(A, B)** Granzyme B (GzmB) expression in lamina propria CD8^+^ T cells or IEL subsets from the small intestine of SPF, GF, and AF mice (*n* = 5 per group). Granzyme B expression was measured without *in vitro* restimulation. **(A)** Representative histograms of granzyme B expression. **(B)** Quantification of granzyme B expression shown as mean fluorescence intensity (MFI) for the indicated IEL subsets. **(C, D)** Splenocytes and small intestinal IELs were harvested from adult SPF, GF, and AF mice (*n* = 6 per SPF and GF mice, *n* = 5 per AF mice). Cells were stimulated *in vitro* for 3 h with PMA/Ionomycin. **(C)** Representative contour plots showing IFN-γ^+^ TCRαβ^+^ CD8αβ^+^ IELs. **(D)** Percentage of IFN-γ^+^ cells gated on TCRαβ^+^ CD8αβ^+^ IELs and total number of IFN-γ^+^ TCRαβ^+^ CD8αβ^+^ IELs. **(E)** Representative dot plots showing IFN-γ^+^ TCRαβ^+^ CD4^+^ IELs. **(D)** Percentage of IFN-γ^+^ cells gated on TCRαβ^+^ CD4^+^ IELs and total number of IFN-γ^+^ TCRαβ^+^ CD4^+^ IELs. In all histograms, granzyme B expression in the indicated cells was shown with thick lines and that in TCRαβ^–^ TCRγδ^–^ cells was shown with dotted lines. Statistical differences were determined by one-way ANOVA **(B, D, F)** with Tukey’s multiple comparisons tests. *p < 0.05, **p < 0.01, ***p < 0.001. ns, not significant. Each symbol represents an individual mouse. Error bars represent SEM.

To precisely determine the influence of gut microbiota- and diet-derived antigens on IEL effector function, we first examined cytolytic activity in conventional IEL subsets from SPF, GF, and AF mice. Granzyme B production was clearly detected in all IEL subsets except for TCRαβ^+^ CD4^+^ IELs (**Figure 2A**). Among granzyme B-producing IELs, levels of intracellular granzyme B were not significantly reduced in GF mice compared to SPF mice (**Figure 2B**). However, in AF mice, granzyme B production was severely impaired across both conventional (TCRαβ^+^ CD8αβ^+^ and TCRαβ^+^ CD4^+^ CD8αα^+^) and unconventional (TCRαβ^+^ CD8αα^+^ and TCRγδ^+^) IELs, indicating that dietary antigens, even if unprocessed by the gut microbiota, are sufficient to induce granzyme B production in IELs (**Figures 2A, B**).

In terms of IFN-γ production, the requirement of gut microbiota and dietary antigens in IFN-γ production varied across conventional IEL subsets. IFN-γ production in conventional TCRαβ^+^ CD8αβ^+^ IELs was partially dependent on gut microbiota, while further deprivation of dietary antigens led to a severe reduction in IFN-γ production in this subset (**Figures 2C, D**). In contrast, IFN-γ production by TCRαβ^+^ CD4^+^ IELs was unaffected in GF mice but was completely abrogated in AF mice (**Figures 2E, F**).

T-bet (T-box expressed in T cells) is a transcription factor previously identified in certain IEL subsets in normal SPF mice (25, 26). While other transcription factors such as Eomesodermin can promote granzyme B expression, T-bet acts as a potent enhancer of granzyme B expression (27, 28). Consistent with the diminished granzyme B expression, T-bet was significantly reduced in conventional TCRαβ^+^ CD8αβ^+^ IELs in AF mice compared to SPF and GF mice (**Supplementary Figures S1A, B**). Likewise, T-bet expression was also downregulated in unconventional TCRαβ^+^ CD8αα^+^ and TCRγδ^+^ IELs from AF mice relative to those in SPF and GF mice (**Supplementary Figures S1C, D**). Flow cytometry and qPCR analyses further revealed that Blimp1 expression was significantly reduced in TCRαβ^+^ CD8αβ^+^ IELs from AF mice compared to GF mice (**Supplementary Figures S1E, F**), in line with their tissue localization patterns (29). Together, these findings highlight that dietary antigens are critical for optimal IEL effector function, at least in part, through the regulation of transcription factors such as T-bet and Blimp-1.

### Generation and maintenance of functional IELs require stimulation with dietary antigens

**Figure 3:**
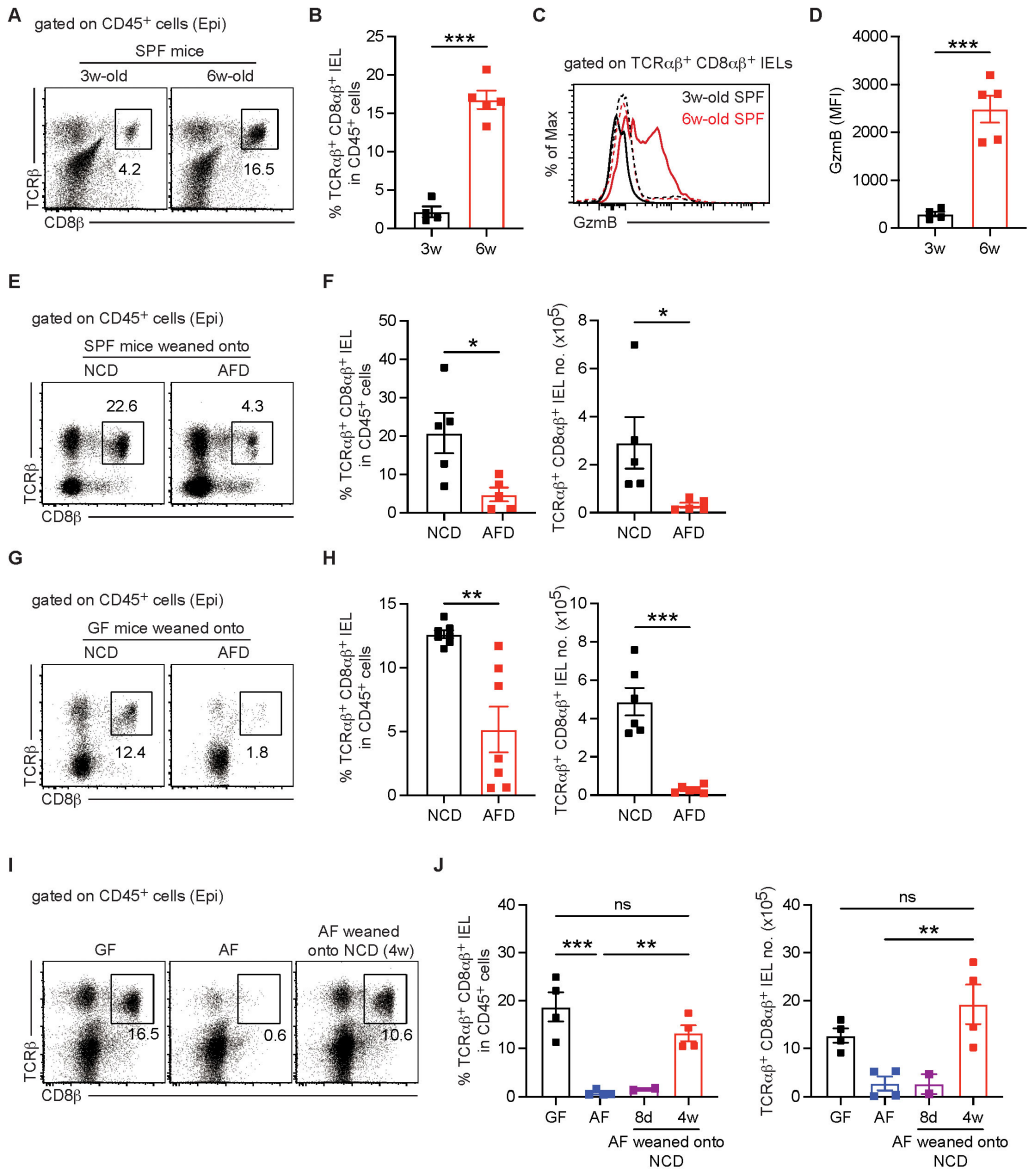
Exposure to diet during ontogeny drives IEL development and effector functions. IELs were harvested from the small intestine of the indicated mice and TCRαβ^+^ CD8αβ^+^ T cells were identified by staining with anti-TCRβ and CD8β mAbs. **(A–D)** TCRαβ^+^ CD8αβ^+^ IELs were examined in 3- and 6-week-old SPF mice (*n* = 4–5 per group). **(A)** Representative dot plots showing TCRαβ^+^ CD8αβ^+^ IELs gated on CD45^+^ cells. **(B)** Percentage of TCRαβ^+^ CD8αβ^+^ IELs gated on CD45^+^ cells. **(C)** Representative histogram showing granzyme B expression in TCRαβ^+^ CD8αβ^+^ IELs. Granzyme B expression in the indicated cells was shown with thick lines and that in TCRβ^–^ TCRγδ^–^ cells was shown with dotted lines. **(D)** MFI of granzyme B expression in TCRαβ^+^ CD8αβ^+^ IELs. **(E, F)** Three-week-old SPF mice were weaned onto normal chow diet (NCD) or antigen-free diet (AFD) and TCRαβ^+^ CD8αβ^+^ IELs were examined at 3 weeks post-weaning (*n* = 5 per group). **(E)** Representative dot plots gated on showing TCRαβ^+^ CD8αβ^+^ IELs gated on CD45^+^ cells. **(F)** Percentage of TCRαβ^+^ CD8αβ^+^ IELs gated on CD45^+^ cells (left) and total numbers of TCRαβ^+^ CD8αβ^+^ IELs (right). **(G, H)** Three-week-old GF mice were weaned onto NCD and AFD, and TCRαβ^+^ CD8αβ^+^ IELs were examined at 3 weeks post-weaning (*n* = 7 per group). **(G)** Representative dot plots gated on showing TCRαβ^+^ CD8αβ^+^ IELs gated on CD45^+^ cells. **(H)** Percentage of TCRαβ^+^ CD8αβ^+^ IELs gated on CD45^+^ cells (left) and total numbers of TCRαβ^+^ CD8αβ^+^ IELs (right). **(I, J)** Three-week-old AF mice were weaned onto NCD, and TCRαβ^+^ CD8αβ^+^ IELs were examined 8 days and 4 weeks later after weaning (*n* = 4 per GF, AF, and AF mice weaned onto NCD for 4 weeks, *n* = 2 per AF mice weaned onto NCD for 8 days). **(I)** Representative dot plots gated on showing TCRαβ^+^ CD8αβ^+^ IELs gated on CD45^+^ cells in the indicated mice. **(J)** Percentage of TCRαβ^+^ CD8αβ^+^ IELs gated on CD45^+^ cells (left) and total numbers of TCRαβ^+^ CD8αβ^+^ IELs (right) in the indicated mice. At least two independent experiments show similar results. Statistical differences were determined by unpaired Student’s *t*-test **(F, H)** or by one-way ANOVA **(J)** with Tukey’s multiple comparisons tests. *p < 0.05, **p < 0.01, ***p < 0.001. ns, not significant. Each symbol represents an individual mouse. Error bars represent SEM.

Exposure to solid food after weaning and subsequent microbial changes are considered as a critical factor for the development or maturation of the mucosal immune system (30). To investigate the ontogeny of IELs and IEL effector function under physiological conditions, we examined IEL populations in SPF mice before and after weaning onto a normal chow diet (NCD). Conventional TCRαβ^+^ CD8αβ^+^ IELs were scarce in 3-week-old mice but gradually increased following weaning onto NCD (**Figures 3A, B**), consistent with previous findings (1). The proportion of unconventional TCRαβ^+^ CD8αα^+^ IELs among TCRαβ^+^ CD8α^+^ cells was relatively higher in pre-weaned SPF mice compared to adults, reflecting the age-dependent expansion of conventional TCRαβ^+^ CD8αβ^+^ IELs (**Supplementary Figure S2A**). TCRγδ^+^ IELs also increased after weaning onto NCD (**Supplementary Figure S2B**). Furthermore, upon weaning onto NCD, granzyme B expression was substantially upregulated in both conventional TCRαβ^+^ CD8αβ^+^ IELs and unconventional TCRαβ^+^ CD8αα^+^ and TCRγδ^+^ IELs (**Figures 3C, D**, and **Supplementary Figures S2A, B**).

To further confirm that dietary antigens drive the IEL development, neonatal SPF or GF mice were weaned onto either NCD or AF diet (AFD). Regardless of the presence of gut microbiota, both SPF and GF mice weaned onto an AFD for 3 weeks showed a marked depletion of conventional TCRαβ^+^ CD8αβ^+^ IELs (**Figures 3E–H**). In contrast, weaning neonatal SPF or GF mice onto NCD for 3 weeks did not reveal a significant effect of AFD feeding on the frequency or total number of unconventional TCRαβ^+^ CD8αα^+^ IELs (**Supplementary Figures S2C, E**). Consistent with the levels of TCRγδ^+^ IELs observed in AF mice (**Figure 1H**), the abundance of TCRγδ^+^ IELs was similar between mice weaned onto NCD and those weaned onto AFD (**Supplementary Figures S2D, F**). Conversely, we examined IEL development in neonatal AF mice weaned onto a sterile NCD. Weaning neonatal AF mice onto NCD for 4 weeks did not significantly alter the total number of unconventional TCRαβ^+^ CD8αα^+^ IELs (**Supplementary Figure S3A**). The abundance of TCRγδ^+^ IELs remained similar among GF, AF, and AF mice weaned onto NCD (**Supplementary Figure S3B**). However, pre-weaned AF mice maintained onto NCD for 4 weeks developed TCRαβ^+^ CD8αβ^+^ IELs at levels similar to GF mice (**Figures 3I, J**), accompanied by an increase in granzyme B expression within this subset (**Supplementary Figure S3C**). Granzyme B expression in unconventional IEL subsets was restored to the level observed in GF mice (**Supplementary Figure S3C**).

Based on these findings, we also tested whether deprivation of dietary antigens leads to the impairment of TCRαβ^+^ CD8αβ^+^ IEL generation by using commercially available AAD, which are depleted of proteins, as reported previously (16, 31). Weaning neonatal GF mice onto both AFD and AAD effectively prevented the development of IELs, particularly TCRαβ^+^ CD8αβ^+^ IELs (**Supplementary Figures S4A, B**). In addition, granzyme B expression on the residual IELs that developed in AAD-fed GF mice was very low (**Supplementary Figure S4C**). Collectively, these findings indicate that dietary antigens exposed during the weaning period are essential for both the development and functional maturation of IELs.

### Continuous stimulation with dietary antigens is required for the effector function of IELs

**Figure 4:**
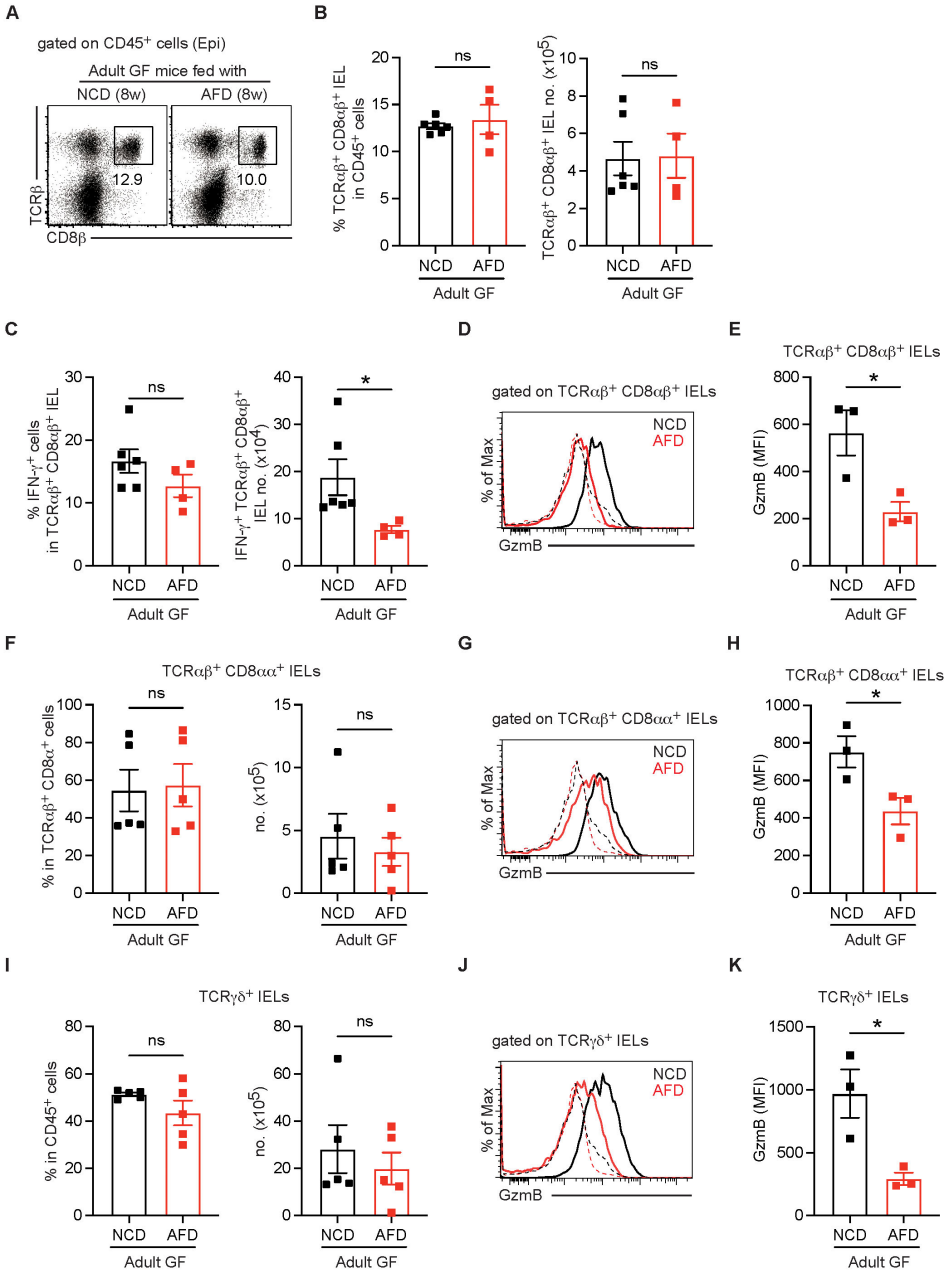
Maintenance of functional IELs requires stimulation with dietary antigens. Adult GF mice raised on sterile NCD were kept fed with NCD or switched to AFD for 8 weeks and TCRαβ^+^ CD8αβ^+^ IELs in the small intestine were examined (*n* = 6 per adult GF mice fed with NCD, *n* = 4 per adult GF mice fed with AFD). **(A)** Representative dot plots gated on showing TCRαβ^+^ CD8αβ^+^ IELs gated on CD45^+^ cells. **(B)** Percentage of TCRαβ^+^ CD8αβ^+^ IELs gated on CD45^+^ cells (left) and total numbers of TCRαβ^+^ CD8αβ^+^ IELs (right). **(C)** Percentage of IFN-γ^+^ cells gated on TCRαβ^+^ CD8αβ^+^ IELs (left) and total number of IFN-γ^+^ TCRαβ^+^ CD8αβ^+^ IELs (right). **(D)** Representative histogram showing granzyme B expression in TCRαβ^+^ CD8αβ^+^ IELs. **(E)** MFI of granzyme B expression in TCRαβ^+^ CD8αβ^+^ IELs from the indicated mice (*n* = 3 per group). **(F)** Percentage of TCRαβ^+^ CD8αα^+^ IELs gated on TCRαβ^+^ CD8α^+^ IELs (left) and total number of TCRαβ^+^ CD8αα^+^ IELs (right). **(G)** Representative histogram showing granzyme B expression in TCRαβ^+^ CD8αα^+^ IELs. **(H)** MFI of granzyme B expression in TCRαβ^+^ CD8αα^+^ IELs from the indicated mice (*n* = 3 per group). **(I)** Percentage of TCRγδ^+^ IELs gated on CD45^+^ cells (left) and total number of TCRγδ^+^ IELs (right). **(J)** Representative histogram showing granzyme B expression in TCRγδ^+^ IELs. **(H)** MFI of granzyme B expression in TCRγδ^+^ IELs from the indicated mice (*n* = 3 per group). Data are pooled from two independent experiments **(B, C, F, I)**. Two independent experiments show similar results **(E, H, K)**. In all histograms, granzyme B expression in the indicated cells was shown with thick lines and that in TCRβ^–^ TCRγδ^–^ cells was shown with dotted lines. Statistical differences were determined by unpaired Student’s *t*-test. *p < 0.05, ns, not significant. Each symbol represents an individual mouse. Error bars represent SEM.

Next, to investigate whether continued exposure to dietary antigens is required for maintaining conventional IEL subsets, adult GF mice with a full IEL compartment were fed with AFD for 8 weeks. Surprisingly, we found that the proportion and total number of conventional TCRαβ^+^ CD8αβ^+^ IELs remained stable despite AFD feeding (**Figures 4A, B**). However, their effector function, as measured by IFN-γ production and granzyme B expression, was significantly impaired by AFD feeding (**Figures 4C–E**). AFD feeding to adult GF mice also led to the impaired effector function in unconventional IELs, such as TCRαβ^+^ CD8αα^+^ and TCRγδ^+^ IEL subsets, although both IEL populations were also long-lived (**Figures 4F–K**). These findings suggest that while IELs are long-lived, their effector function requires continued stimulation by dietary antigens.

### IL-12 can sustain granzyme B expression in TCRαβ^+^ CD8αβ^+^ IELs even in the absence of dietary antigen stimulation

While continuous stimulation with dietary antigens is critical for maintaining the effector function of both conventional and unconventional IELs, intestinal cytokine milieu influenced by dietary macromolecules may also regulate their effector function. Previous studies have reported that intestinal epithelium shows high expression of IL-15 and TGF-β (32, 33) and that IELs exert cytolytic activity upon exposure to IL-12, IL-18, and IL-15 (34). To address this issue, in subsequent analyses, we focused on conventional TCRαβ^+^ CD8αβ^+^ IELs to avoid the complexity and heterogeneity associated with unconventional IEL subsets. Moreover, TCRαβ^+^ CD8αβ^+^ IELs can be effectively studied by using experimental approaches such as adoptive transfer of TCR-transgenic CD8^+^ T cells combined with feeding of cognate antigen as a model dietary antigen.

**Figure 5:**
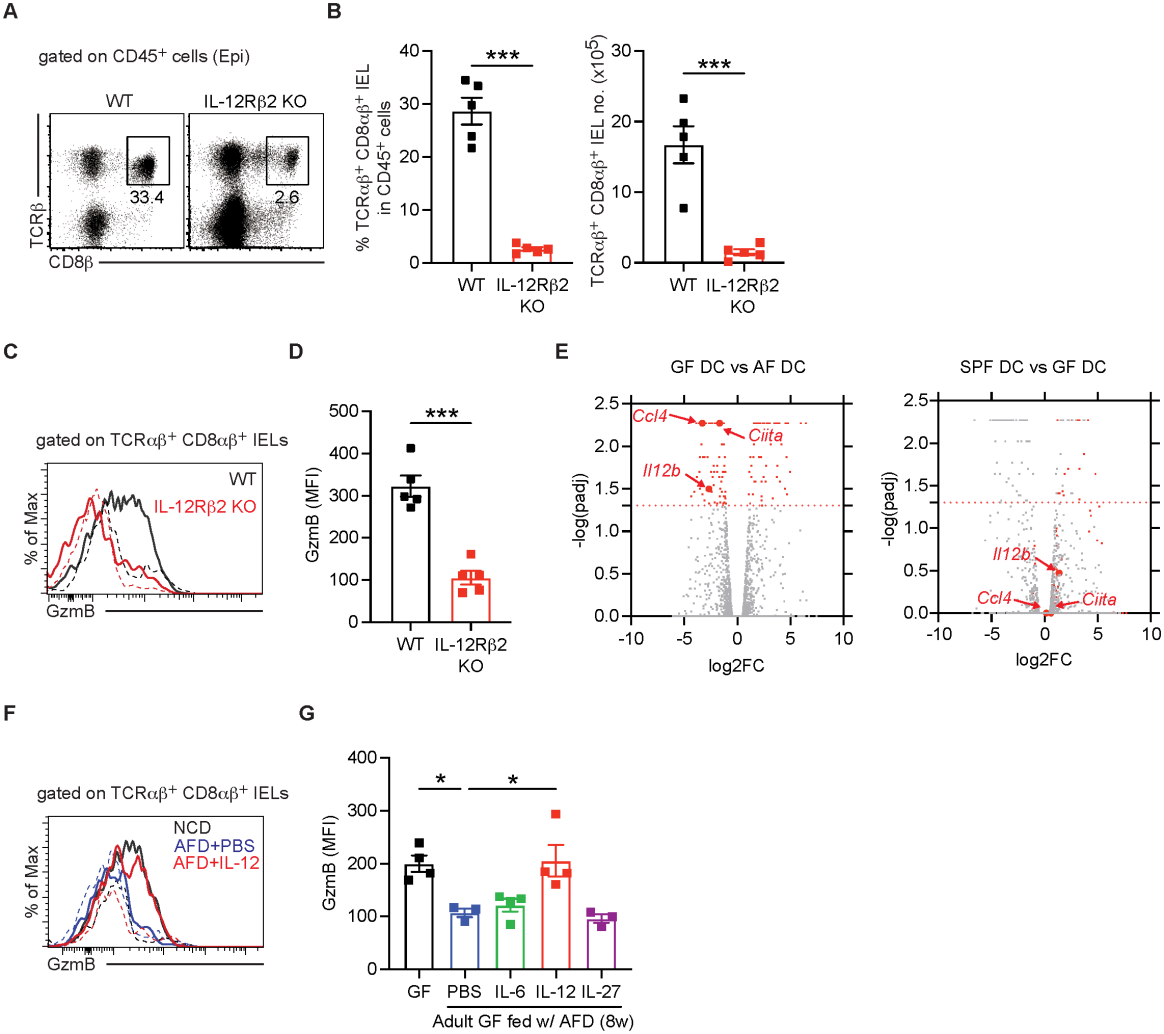
IL-12 can sustain granzyme B expression in TCRαβ^+^ CD8αβ^+^ IELs in the absence of dietary antigen stimulation. **(A–D)** IELs were harvested from the small intestine of adult SPF B6 wild-type (WT) and IL-12Rβ2 knockout (KO) mice and TCRαβ^+^ CD8αβ^+^ IELs were analyzed (*n* = 5 per group). **(A)** Representative dot plots gated on showing TCRαβ^+^ CD8αβ^+^ IELs gated on CD45^+^ cells. **(B)** Percentage of IFN-γ^+^ cells gated on TCRαβ^+^ CD8αβ^+^ IELs (left) and total number of IFN-γ^+^ TCRαβ^+^ CD8αβ^+^ IELs (right). **(C)** MFI of granzyme B expression in TCRαβ^+^ CD8αβ^+^ IELs from the indicated mice. **(D)** MFI of granzyme B expression in TCRαβ^+^ CD8αβ^+^ IELs. **(E)** CD103^+^ CD11b^+^ dendritic cells (DCs) were FACS-sorted from the lamina propria of the small intestine of adult SPF, GF, and AF mice. Gene expression was analyzed by RNA-seq. Volcano plots show differentially expressed genes between GF *vs*. AF mice (left) and SPF *vs*. GF mice (right). Red dots represent genes significantly upregulated in GF *vs*. AF comparison. *Il12b*, *Ccl4*, and *Ciita* were highlighted as representative genes. Red horizontal lines indicate adjusted *p*-value (*p*_adj_) = 0.05. **(F, G)** Adult GF mice were switched to AFD for 8 weeks and then were injected with recombinant IL-6, IL-12, or IL-27 every 3 days for 3 weeks. Granzyme B expression in TCRαβ^+^ CD8αβ^+^ IELs was analyzed. **(F)** Representative histogram showing granzyme B expression in TCRαβ^+^ CD8αβ^+^ IELs. **(G)** MFI of granzyme B expression in TCRαβ^+^ CD8αβ^+^ IELs in the indicated mice. Two independent experiments show similar results. In all histograms, granzyme B expression in the indicated cells was shown with thick lines and that in TCRβ^–^ TCRγδ^–^ cells was shown with dotted lines. Statistical differences were determined by unpaired Student’s *t*-test **(B, D)** or by one-way ANOVA **(G)** with Tukey’s multiple comparisons tests. *p < 0.05, ***p < 0.001. Each symbol represents an individual mouse. Error bars represent SEM.

To determine whether these cytokines contribute to the effector function of dietary antigen-induced IELs, we first analyzed IELs in IL-15-deficient (IL-15KO), CD4-DN TGF-βR2 Tg (CD4-DN TGF-βR2 Tg), and IL-12Rβ2-deficient (IL-12Rβ2 KO) mice. Interestingly, the proportion of conventional TCRαβ^+^ CD8αβ^+^ IELs increased in IL-15KO mice. However, their total numbers and granzyme B expression remained unchanged in IL-15 KO mice (**Supplementary Figures S5A–C**). In CD4-DN TGF-βR2 Tg mice, the levels of TCRαβ^+^ CD8αβ^+^ IELs were significantly reduced, yet granzyme B expression was similar to WT mice (**Supplementary Figures S5E–G**). In contrast, IL-12Rβ2 KO mice exhibited a profound depletion of TCRαβ^+^ CD8αβ^+^ IELs, and the few remaining IELs failed to express granzyme B (**Figures 5A–D**). These results suggest that IL-12 signaling is critical for both the induction of TCRαβ^+^ CD8αβ^+^ IELs and their effector function, whereas either IL-15 or TGF-β signaling is dispensable for their effector function.

Notably, RNA-seq analysis of purified intestinal CD103^+^ CD11b^+^ DCs, the most abundant DCs in the small intestine, revealed that *Il12b* mRNA expression in CD103^+^ CD11b^+^ DCs was similar between SPF and GF mice but significantly reduced in AF mice (**Figure 5E**). Of note, levels of CD103^+^ CD11b^+^ DCs were reduced in AF mice compared to SPF and GF mice (13). To further investigate the role of IL-12 in regulating TCRαβ^+^ CD8αβ^+^ IEL effector function, we examined the effects of injecting innate cytokines, such as IL-6, IL-12, and IL-27, into adult GF mice fed with AFD. Since the effector function of IEL subsets declined following prolonged AFD feeding (**Figures 4A–D**), adult GF mice were given repeated cytokine injections for 3 weeks, starting at 5 weeks after switching the mice onto AFD. Intriguingly, IL-12 injection restored granzyme B expression in TCRαβ^+^ CD8αβ^+^ IELs (**Figures 5F, G**). IL-12 injection did not affect granzyme B expression on splenic and LP-activated CD8^+^ T cells (**Supplementary Figures S6A, B**). Collectively, these data suggest that dietary antigens as well as IL-12, presumably produced by intestinal DCs, are critical for the prolonged function of TCRαβ^+^ CD8αβ^+^ IELs.

### Dietary antigen-induced IELs are required for early protection from foodborne infectious pathogens

**Figure 6:**
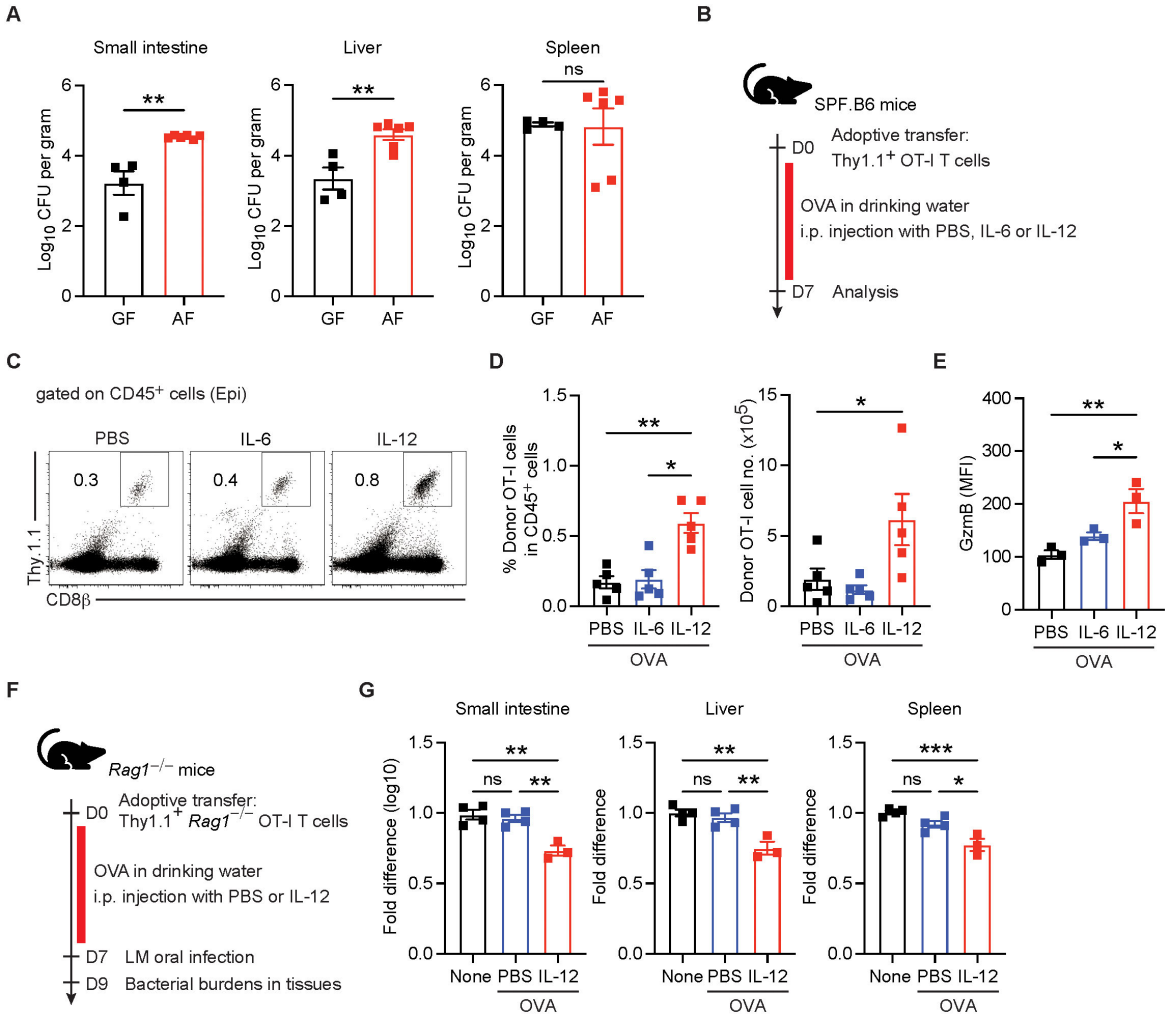
Dietary antigen-induced IELs are required for early protection from foodborne infectious pathogens. **(A)** Adult GF and AF mice were orally infected with *Listeria monocytogenes* expressing mutated internalin A, and bacterial burdens in the indicated tissues, shown as colony-forming unit (CFU), were determined at 2 days post-infection (*n* = 4 per GF mice, *n* = 6 per AF mice). **(B–E)** Adult SPF WT mice were adoptively transferred withThy1.1^+^ OT-I cells and fed with ovalbumin (OVA) for 7 days, together with intraperitoneal injections of PBS and recombinant IL-6 or IL-12 proteins. IELs were harvested from the small intestine of the indicated mice to analyze repopulation of OT-I cells in the small intestinal epithelium. **(B)** Experimental scheme. **(C)** Representative dot plots showing donor OT-I cells. **(D)** Percentage of donor OT-I cells gated on CD45^+^cells (left) and total number of donor OT-I cells (right) in the small intestinal epithelium. **(E)** MFI of granzyme B expression in OT-I IELs from the indicated mice. **(F, G)** Adult SPF *Rag1*^–/–^ mice were adoptively transferred with Thy1.1^+^*Rag1*^–/–^ OT-I cells and fed with normal or OVA-containing drinking water for 1 week. OVA-fed mice were also treated with PBS or recombinant IL-12 proteins every other day for 7 days. Mice were orally infected with *L. monocytogenes* (LM) expressing mutated internalin A and at day 2 post-infection, CFUs were determined in the indicated tissues. None: no OVA feeding, PBS: OVA feeding only, IL-12: OVA feeding + rIL12 injection. **(F)** Experimental scheme. **(G)** Fold differences in bacterial burden among the indicated tissues. Two independent experiments show similar results. Statistical differences were determined by unpaired Student’s *t*-test **(A)** or by one-way ANOVA **(C, E, F)** with Tukey’s multiple comparisons tests. *p < 0.05, **p < 0.01, ***p < 0.001. ns, not significant. Each symbol represents an individual mouse. Error bars represent SEM.

We hypothesized that dietary antigen-induced IELs might provide protection against foodborne pathogens. To assess this idea, we orally infected adult GF and AF mice with LM expressing a mutated internalin A, which enables the bacterium to recognize murine E-cadherin expressed on intestinal epithelial cells in mice (35). Although LM can breach the intestinal epithelial barrier in an internalin A-independent manner (36), we selected this LM strain because it efficiently transverses the intestinal epithelium, where IELs are abundant. Mice were infected with LM via oral gavage to mimic listeriosis, and bacteria burden was assessed by analyzing CFU in the small intestine, liver, and spleen. In line with our hypothesis, we observed that AF mice exhibited a significantly higher infection burdens in the small intestine and liver, but not in spleen, compared to GF mice (**Figure 6A**). These findings suggest that dietary antigen-induced IELs contribute to early and local protection against LM intestinal infection.

To further investigate the role of dietary antigen-induced TCRαβ^+^ CD8αβ^+^ IELs in LM protection, we utilized SPF Rag1 KO mice adoptively transferred with OT-I transgenic CD8^+^ T cells, which are specific to OVA; these mice lack all IEL subsets and OVA feeding allows for the repopulation of OT-I cells in the intestinal epithelium (**Figure 6B**). In addition, we administered IL-12 to examine its role in the induction and effector function of TCRαβ^+^ CD8αβ^+^ IELs in an antigen-specific manner. We found that in Rag1 KO mice adoptively transferred with OT-I CD8^+^ T cells, OVA feeding combined with IL-12 treatment not only enhanced the expansion of OT-I CD8^+^ T cells in the intestinal epithelium (**Figures 6C, D**) but also increased granzyme B production, compared to IL-6 treatment (**Figure 6E**).

Next, we orally infected these mice with LM lacking OVA expression to evaluate whether OT-I CD8^+^ IELs could provide early protection in an antigen-independent manner (**Figure 6F**). Although OVA feeding facilitated the repopulation of OT-I CD8^+^ T cells within the intestinal epithelium, these cells alone failed to provide early protection against LM infection. However, IL-12 treatment significantly reduced LM levels in the small intestine and liver at day 2 post-infection (**Figure 6G**). Collectively, these findings suggest that dietary antigen-induced IELs play a critical role in providing innate-like protective function, thereby contributing to rapid and local protection against foodborne infectious pathogens.

## Discussion

Our work demonstrates that dietary antigens profoundly influence the generation and effector functions of conventional TCRαβ^+^ and unconventional IELs in the small intestine. By comparing SPF, GF, and AF mice that lack exogenous antigens derived from gut microbiota and diet, we report here that dietary antigens effectively induce the majority of conventional IELs, including TCRαβ^+^ CD8αβ^+^, TCRαβ^+^ CD8α^+^ CD4^+^, and TCRαβ^+^ CD4^+^ IELs. Notably, dietary antigen-induced TCRαβ^+^ CD8αβ^+^ IELs provide innate-like function to mediate early protection against foodborne pathogens such as LM. These findings emphasize the critical role of these conventional IELs in mitigating the inherent vulnerability of the small intestine, which relies on a single-layered epithelium for its vital function such as nutrient absorption.

The weaning period represents an immunologically critical window that can imprint long-term susceptibility to inflammatory diseases. In this regard, the weaning reaction to microbiota, which is associated with the induction of RORγt^+^ regulatory T cells, has been identified as a key mechanism for regulating inflammatory responses later in life (37). These regulatory T cells are likely driven by the substantial compositional changes in the gut microbiota induced by the introduction of solid food. Hence, disruption of weaning reactions to microbiota through antibiotic treatment is considered as a risk factor for the development of allergy responses. Interestingly, our findings reveal that dietary antigen exposure, rather than microbial cues, is predominantly responsible for IEL development and effector function acquisition. These processes occur similarly in both SPF and GF mice during the weaning period. Therefore, dietary antigens also play a central role in equipping the small intestinal epithelium, the body’s frontline barrier in the intestine, with cytotoxic T cells during the natural weaning period.

Weaning neonatal GF mice onto AFD resulted in the depletion of conventional TCRαβ^+^ CD8αβ^+^ IELs. However, when dietary antigen deprivation occurs later in life, after TCRαβ^+^ CD8αβ^+^ IELs have populated the small intestinal epithelium, their numbers remain unaffected. This suggests that dietary antigen stimulation is not required for the long-term survival of TCRαβ^+^ CD8αβ^+^ IELs as they can persist for extended periods without continued antigen exposure. Considering that, in the context of infections, TCRαβ^+^ CD8αβ^+^ IELs can persist in the intestinal epithelium as tissue-resident memory T (Trm) cells (38, 39), our data suggest that dietary antigen-induced TCRαβ^+^ CD8αβ^+^ IELs may also persist as Trm cells within the epithelium in the absence of dietary antigen exposure. IL-7 signaling is critical for the maintenance of TCRαβ^+^ CD8αβ^+^ IELs as well as Trm cells (40). Hence, IL-7 produced by intestinal epithelial cells might support the survival of these IELs in the absence of antigenic stimulation (41, 42).

Nevertheless, our findings indicate that dietary antigen deprivation functionally impairs conventional TCRαβ^+^ CD8αβ^+^ IELs, with a significant reduction in their cytotoxic activity. While previous studies suggested that TCR engagement is required for IEL differentiation but not effector function (38), our data suggest a refinement to this view. We demonstrate that dietary antigen-specific IELs, particularly TCRαβ^+^ CD8αβ^+^ IELs, require continuous exposure to dietary antigens to maintain optimal function as seen in adult GF mice fed with AFD. Furthermore, dietary antigen-induced TCRαβ^+^ CD8αβ^+^ IELs encompass their activity against LM expressing unrelated antigens. Although it remains elusive how IELs reduce LM burden in an antigen-independent manner and whether IELs target both intracellular and extracellular LM, our results highlight that the poised activation state of TCRαβ^+^ CD8αβ^+^ IELs in response to dietary antigens and innate-like function in a TCR-independent manner contributes to rapid protection against foodborne pathogens in the small intestine. Indeed, TCRαβ^+^ CD8αβ^+^ IELs exhibit an innate-like function through the expression of antimicrobial peptides (43) and NK cell-activating receptors (38).

In addition to the critical role of dietary antigen stimulation, our data suggest that IL-12, presumably produced by small intestinal LP-DCs, is important for both maintenance and effector function of TCRαβ^+^ CD8αβ^+^ IELs. In this regard, defective IL-12 signaling, as seen in IL-12Rβ2 KO mice, results in the reduction of these IELs. Conversely, exogenous IL-12 administration enhances the repopulation of dietary antigen-induced TCRαβ^+^ CD8αβ^+^ IELs and their cytotoxic function, thereby improving the resistance to LM. Interestingly, IL-12 production by LP-DCs remains intact in GF mice but is significantly reduced in AF mice, suggesting that gut microbiota is dispensable for IL-12 production by LP-DCs. It is possible that unknown dietary macromolecules condition LP-DCs to produce IL-12, or alternatively, T cell-intrinsic factors such as CD40L may induce IL-12 production by LP-DCs. The latter scenario can be supported by the observation that both conventional IELs and effector/memory phenotype LP T cells are significantly reduced in AF mice (13). Further investigation is required to elucidate the precise mechanisms governing IL-12 production by LP-DCs.

Previously, unconventional IELs, such as TCRαβ^+^ CD8αα^+^ and TCRγδ^+^ IELs, were shown to possess innate-like properties and to protect foodborne pathogens (44, 45). Our findings suggest that conventional TCRαβ^+^ CD8αβ^+^ IELs also display innate-like and antigen-independent functions in the immune defense against foodborne pathogens. In addition, our results provide new insight into how unconventional IEL functions are regulated. Although the development of TCRαβ^+^ CD8αα^+^ and TCRγδ^+^ IELs is less dependent on dietary antigens than that of conventional TCRαβ^+^ CD8αβ^+^ IELs, deprivation of dietary macromolecules, including dietary antigens, diminishes their cytotoxic activity. Notably, dietary antigens are not required for the induction of TCRγδ^+^ IELs. However, their cytotoxic function is markedly impaired in GF mice fed with AFD. Together, these findings highlight the essential role of dietary macromolecules in maintaining gut immune homeostasis and also underscore the need for future studies to elucidate how dietary components regulate the cytotoxic programming of TCRγδ^+^ T cells.

In summary, our findings establish that dietary antigens play a crucial role in the development and functional maintenance of conventional IELs, which provide early defense against foodborne pathogens. Continuous dietary antigen exposure is essential for sustaining IEL cytotoxic activity, underscoring the relationship between diet and intestinal immune protection. Our data also suggest that malnutrition with severe dietary protein deficiency, as seen in regions affected by famine, may lead to increased susceptibility to foodborne pathogenic infections due to compromised IEL functions.

## Data Availability

Data have been deposited at the National Center for Biotechnology Information, and are publicly available under accession number GSE299783.
