# Supplementary material for: Dietary antigens drive the generation of functional cytotoxic intraepithelial lymphocytes for early defense against foodborne pathogens
Source: Front Immunol. 2026 Jan 7;16:1639120. doi: 10.3389/fimmu.2025.1639120 (PMC12819662; doi:10.3389/fimmu.2025.1639120)
Supplement: Supplementary file 1 [file DataSheet1.zip › Supplementary figures.pdf]

# Supplementary figures

## Dietary antigens drive the generation of functional cytotoxic intraepithelial lymphocytes for early defense against foodborne pathogens

**Jisun Jung,[1,2,3] Jaeu Yi,[1,2,4,*] Kwang Soon Kim,[1,2,*] and Charles D. Surh[1,2,5,6]**

[1]Department of Life Sciences, Pohang University of Science and Technology (POSTECH), Pohang, Republic of Korea

[2]Academy of Immunology and Microbiology, Institute for Basic Science (IBS), Pohang 37673, Republic of Korea

[3]Department of Internal Medicine, Division of Rheumatology, Washington University School of Medicine, St. Louis, MO 63110 MO, United States

[4]Department of Biological Science, Ajou University, Suwon, Republic of Korea

[5]Division of Developmental Immunology, La Jolla Institute for Allergy and Immunology (LIAI), La Jolla, CA 92037, USA

[6]Deceased

*Correspondence: Kwang Soon Kim: kskim27@postech.ac.kr ;Jaeu Yi: jaeuyi@ajou.ac.kr

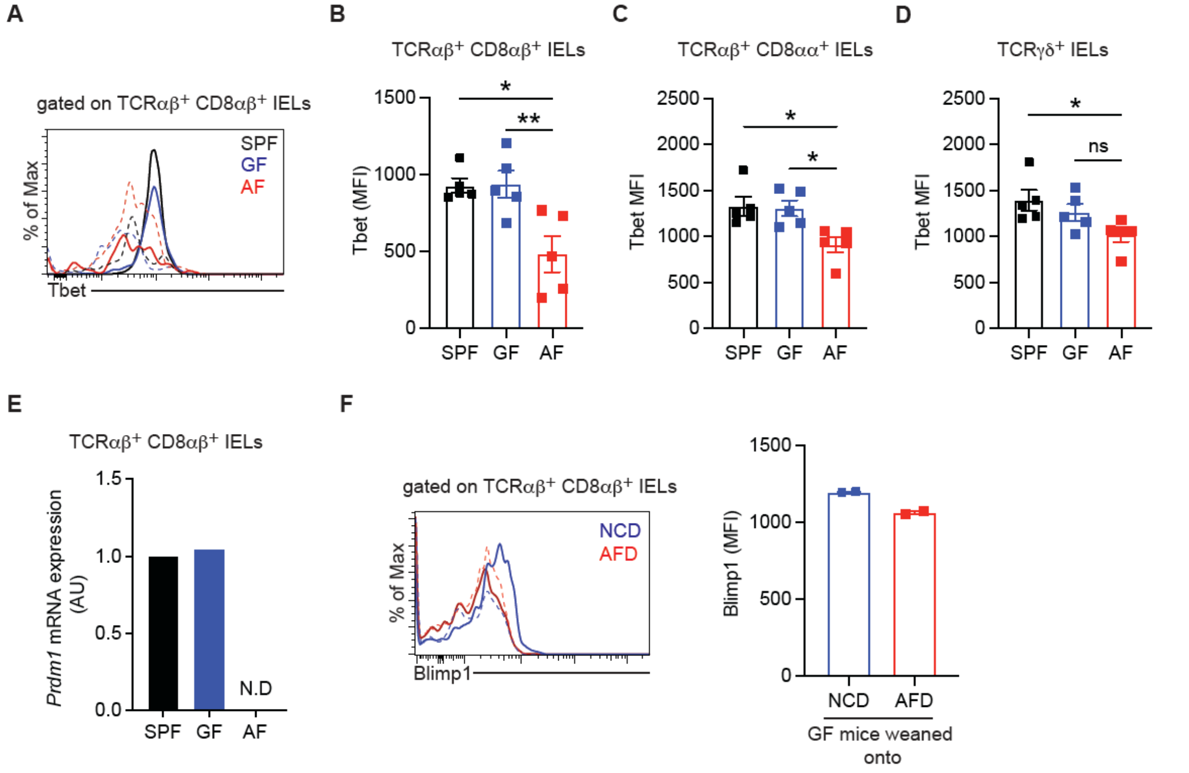


**Figure S1. Transcription factor T-bet and Blimp 1 in intestinal IELs are diminished in AF mice, related to Figure 2.**

(A) Representative histogram showing T-bet expression in $TCR\alpha\beta^{+}$ $CD8\alpha\beta^{+}$ IELs from the small intestine of SPF, GF and AF mice (n=5 per group). (B-D) MFI of Tbet expression in $TCR\alpha\beta^{+}CD8\alpha\beta^{+}$ IELs (B), $TCR\alpha\beta^{+}CD8\alpha\alpha^{+}$ IELs (C) and $TCR\gamma\delta^{+}$ IELs (D). (E) Relative mRNA levels of Blimp1 (*Prdm1*) in sorted small intestinal $TCR\alpha\beta^{+}$ $CD8\alpha\beta^{+}$ IELs from adult SPF, GF and AF mice. (F) Representative histogram showing Blimp1 expression in GF mice weaned onto NCD and AFD (left) and MFI of Blimp1 expression in $TCR\alpha\beta^{+}CD8\alpha\beta^{+}$ IELs (right). Tbet or Blimp1 expression in the indicated cells was shown with thick lines and that in $TCR\beta^{-}$ $TCR\gamma\delta^{-}$ cells was shown with dotted lines. Statistical differences were determined by one-way ANOVA with Tukey's multiple comparisons tests (B). **p*<0.05, ***p*<0.01. ns, not significant. Each symbol represents an individual mouse. Error bars represent SEM.

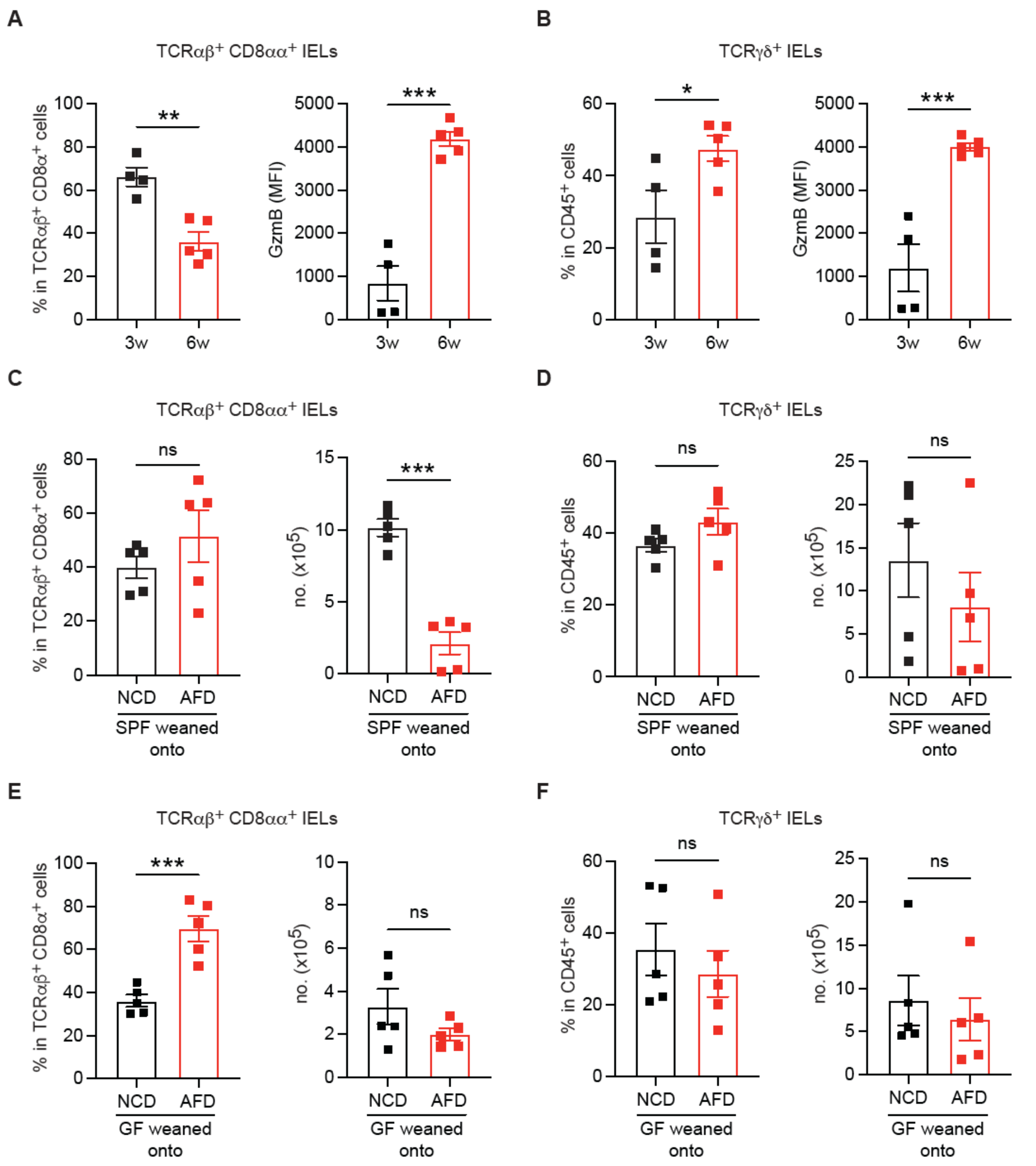


**Figure S2. Influence of dietary antigen deprivation on unconventional IELs, related to Figure 3.**

(A) Percentage of TCRαβ$^+$ CD8αα$^+$ IELs gated on TCRαβ$^+$ CD8α$^+$ IELs (left) and granzyme B expression within this subset (right) in 3-week- and 6-week-old SPF mice (n=4-5 per group). (B) Percentage of TCRγδ$^+$ IELs gated on CD45$^+$ cells (left) and granzyme B expression within this subset (right) in 3-week- and 6-week-old SPF mice (n=4-5 per group). (C, D) 3-week-old SPF mice were weaned onto normal chow diet (NCD) or antigen-free diet (AFD) (n=5 per group). (C) Percentage of

TCRαβ$^+$ CD8αα$^+$ IELs gated on TCRαβ$^+$ CD8α$^+$ IELs (left) and total number of TCRαβ$^+$ CD8αα$^+$ IELs (right). (D) Percentage of TCRγδ$^+$ IELs gated on CD45$^+$ cells (left) and total number of TCRγδ$^+$ IELs (right). (E, F) 3-week-old GF mice were weaned onto NCD and AFD (n=5 per group). (E) Percentage of TCRαβ$^+$ CD8αα$^+$ IELs gated on TCRαβ$^+$ CD8α$^+$ IELs (left) and total number of TCRαβ$^+$ CD8αα$^+$ IELs (right). (F) Percentage of TCRγδ$^+$ IELs gated on CD45$^+$ cells (left) and total number of TCRγδ$^+$ IELs (right). Statistical differences were determined by unpaired Student's t test. *$p$<0.05, **$p$<0.01, ***$p$<0.001, ns, not significant. Each symbol represents an individual mouse. Error bars represent SEM.

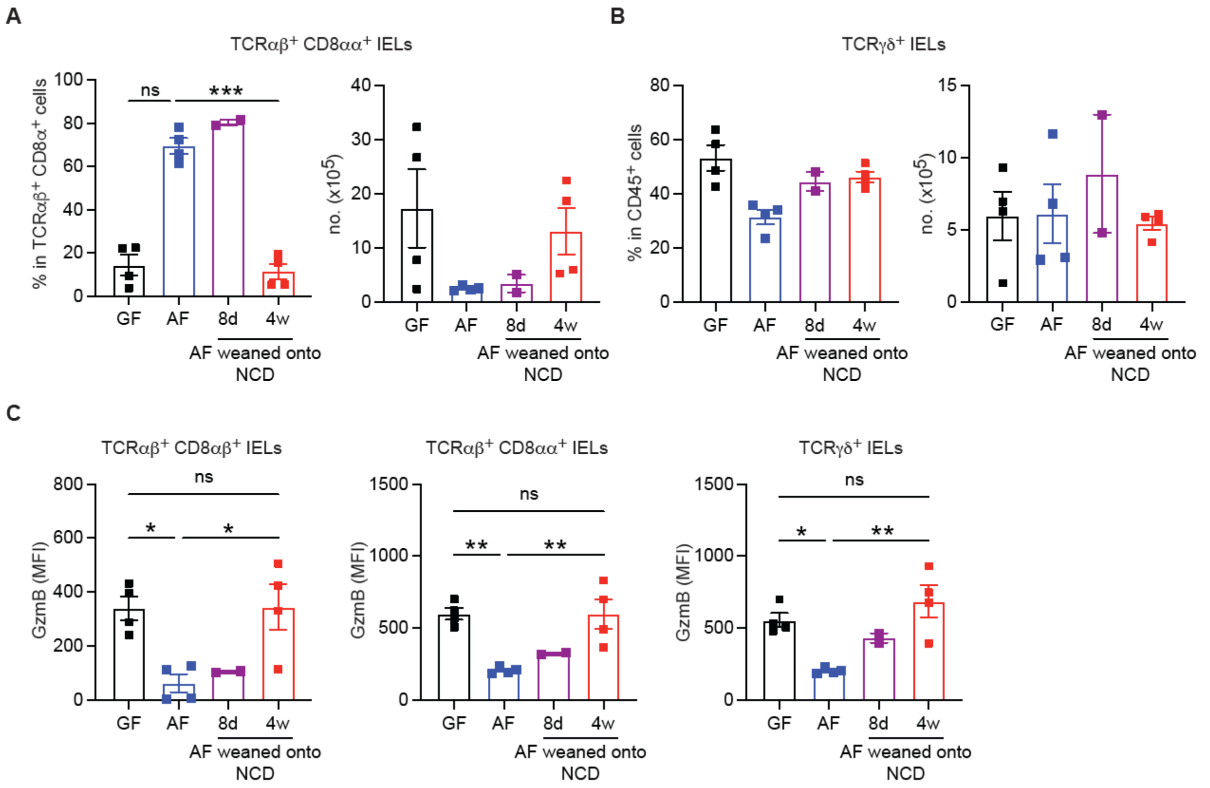


**Figure S3. Influence of dietary antigen exposure on the development and function of IEL subsets in AF mice weaned onto normal chow diet, related to Figure 3.**

3-week-old AF mice were weaned onto NCD, and IEL subsets were examined 8 days and 4 weeks later after weaning (n=4 per GF, AF and AF mice weaned onto NCD for 4 weeks, n=2 per AF mice weaned onto NCD for 8 days). (A) Percentage of TCRαβ$^+$ CD8αα$^+$ IELs gated on TCRαβ$^+$ CD8α$^+$ IELs (left) and total number of TCRαβ$^+$ CD8αα$^+$ IELs (right). (B) Percentage of TCRγδ$^+$ IELs gated on CD45$^+$ cells (left) and total number of TCRγδ$^+$ IELs (right). (C) MFI of granzyme B expression in TCRαβ$^+$ CD8αβ$^+$ IELs (left), TCRαβ$^+$ CD8αα$^+$ IELs (middle) and TCRγδ IELs (right). Statistical differences were determined by one-way ANOVA with Tukey's multiple comparisons tests (B). *$p$<0.05, **$p$<0.01, ***$p$<0.001. ns, not significant. Each symbol represents an individual mouse. Error bars represent SEM.

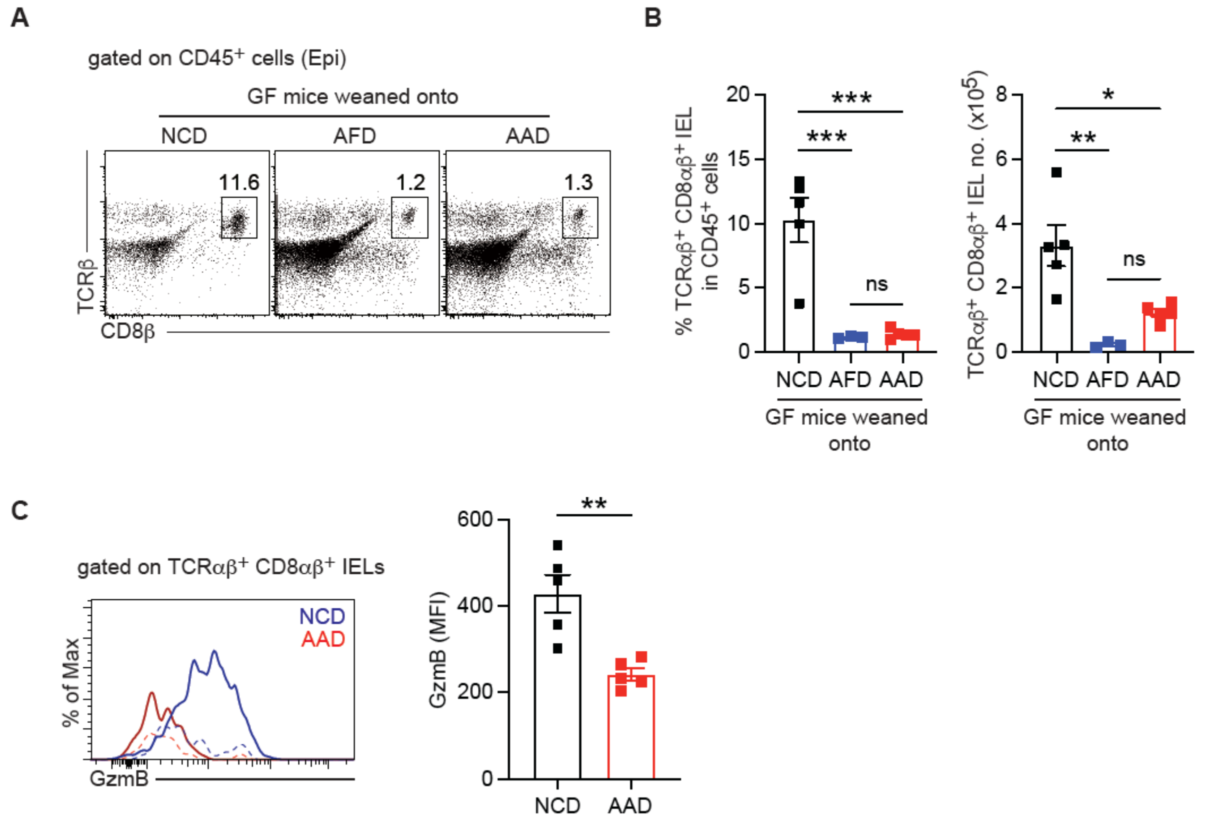


**Figure S4. Impaired generation and functions of TCRαβ⁺ CD8αβ⁺ IELs in GF mice weaned onto AFD and commercial amino-acid diet.**

Neonatal GF mice weaned onto normal chow diet (NCD), antigen-free diet (AFD) and commercially available amino-acid diet (AAD) for 4 weeks. (A) Representative dot plots showing TCR$\alpha\beta^+$ CD8$\alpha\beta^+$ IELs gated on CD45$^+$ cells in the small intestine from the indicated mice. (B) Percentage of TCR$\alpha\beta^+$ CD8$\alpha\beta^+$ IELs gated on CD45$^+$ cells (left) and total number of TCR$\alpha\beta^+$ CD8$\alpha\beta^+$ IELs (right). (C) Representative histogram showing granzyme B expression (left) and MFI of granzyme B expression (right) in the indicated mice. Granzyme B expression in the indicated cells was shown with thick lines and that in TCR$\beta^-$ TCR$\gamma\delta^-$ cells was shown with dotted lines. Statistical differences were determined by unpaired Student's *t* test (B) or one-way ANOVA (C) with Tukey's multiple comparisons tests. *$p$<0.05, **$p$<0.01, ***$p$<0.001. ns, not significant. Each symbol represents an individual mouse. Error bars represent SEM.

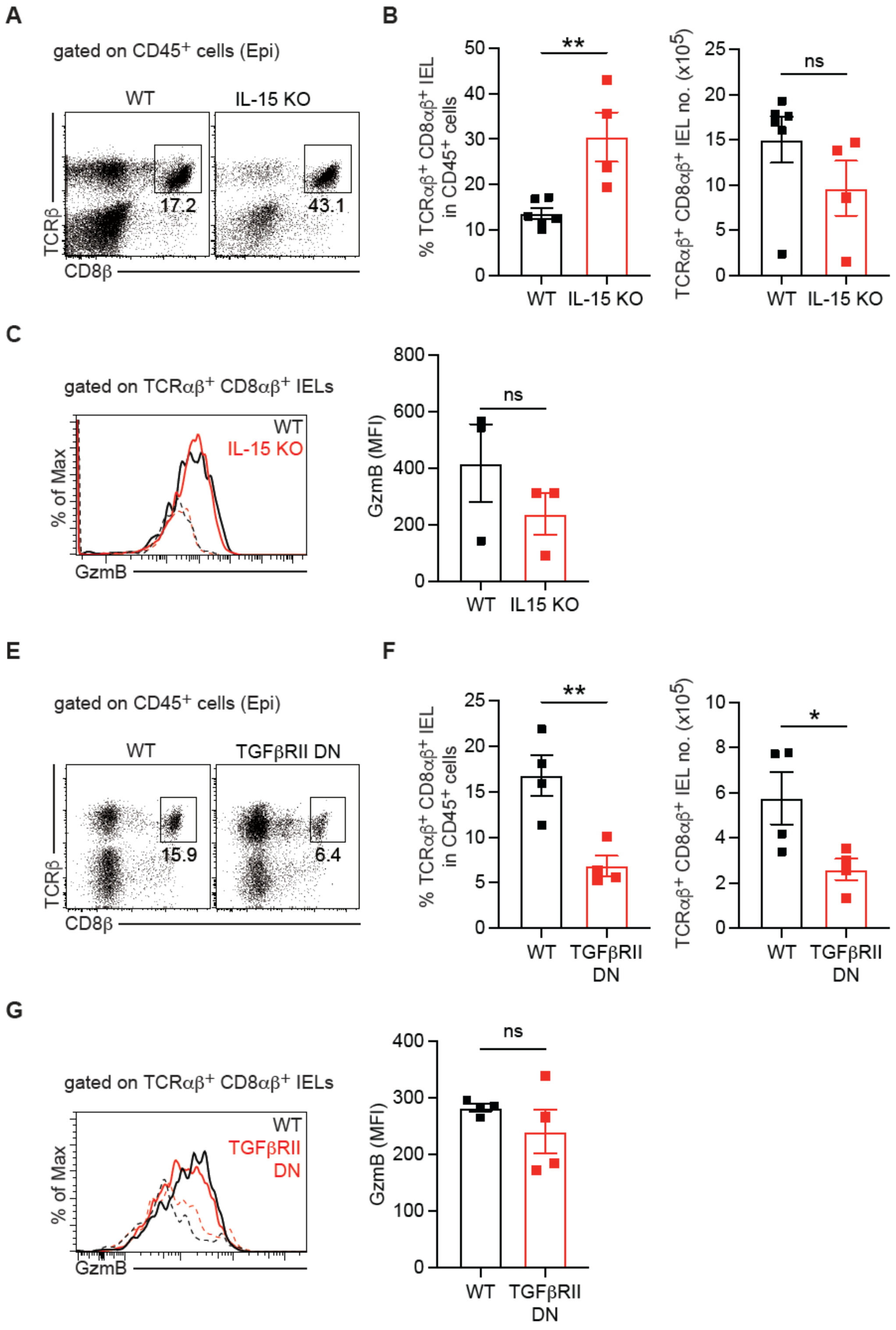


**Figure S5. Both TGFβ and IL-15 are not required for CD8αβ⁺ IELs to express granzyme B, related to Figure 5**

(A-C) Small intestinal CD8$\alpha\beta^+$ IELs were compared in B6 WT and IL15 KO mice. (A) Representative dot plots showing TCR$\alpha\beta^+$ CD8$\alpha\beta^+$ IELs gated on CD45$^+$ cells. (B) Percentage of TCR$\alpha\beta^+$ CD8$\alpha\beta^+$ IELs gated on CD45$^+$ cells (left) and total number of TCR$\alpha\beta^+$ CD8$\alpha\beta^+$ IELs (right). (C) Representative histogram showing granzyme B expression (left) and MFI of granzyme B expression (right) in the indicated mice. (E-G) Small intestinal CD8$\alpha\beta^+$ IELs were compared in B6 WT and CD4-dominant negative (DN) TGF$\beta$RII Tg mice. (E) Representative dot plots showing TCR$\alpha\beta^+$ CD8$\alpha\beta^+$ IELs gated on CD45$^+$ cells. (F) Percentage of TCR$\alpha\beta^+$ CD8$\alpha\beta^+$ IELs gated on CD45$^+$ cells (left) and total number of TCR$\alpha\beta^+$ CD8$\alpha\beta^+$ IELs (right). (G) Representative histogram showing granzyme B expression (left) and MFI of granzyme B expression (right) in the indicated mice. In all histograms, granzyme B expression in the indicated cells was shown with thick lines and that in TCR$\beta^-$ TCR$\gamma\delta^-$ cells was shown with dotted lines. Statistical differences were determined by unpaired Student's *t* test. *$p<0.05$, **$p<0.01$. ns, not significant. Each symbol represents an individual mouse. Error bars represent SEM.

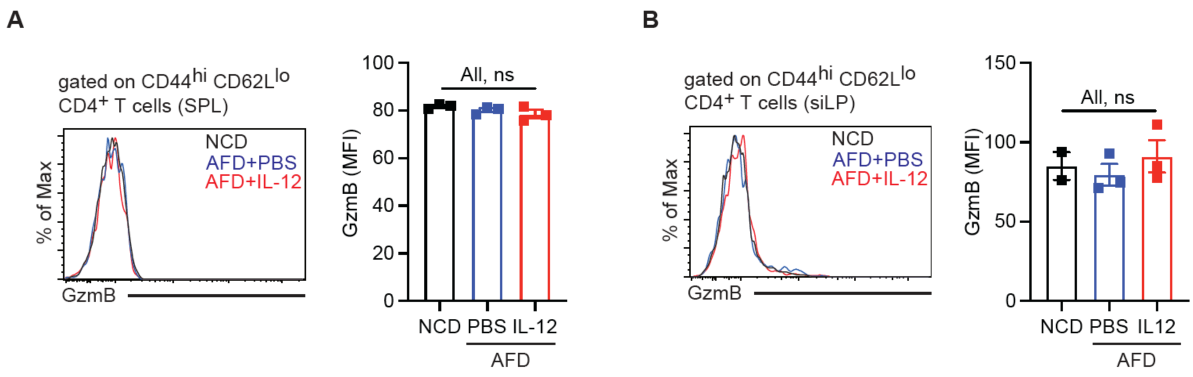


**Figure S6. Exogenous IL-12 do not enhance granzyme B expression on activated CD8 T cells in the spleen and lamina propria, related to Figure 5.**

Adult GF mice were switched to AFD for 8 weeks, and were injected with recombinant IL-12 every three days for the last 3 weeks. Activated CD8$^{+}$ T cells from the spleen (A) or small intestinal lamina propria (B) were analyzed for their granzyme B expression, which were shown by representative histograms of granzyme B expression (left) and MFI of granzyme B expression (right). Statistical differences were determined by unpaired Student's *t* test (B) or one-way ANOVA (C) with Tukey's multiple comparisons tests. ns, not significant. Each symbol represents an individual mouse. Error bars represent SEM.
